# ACSL4 mediates inflammatory bowel disease and contributes to LPS-induced intestinal epithelial cell dysfunction by activating ferroptosis and inflammation

**DOI:** 10.1515/med-2024-0993

**Published:** 2024-09-03

**Authors:** Ieng-Hou Lam, Chon-In Chan, Meixia Han, Lixuan Li, Hon-Ho Yu

**Affiliations:** Department of Gastroenterology, Kiang Wu Hospital, Macau, SAR 999078, China; Department of Gastroenterology, Guangdong Second Provincial General Hospital, Guangzhou, 510000, Guangdong Province, China

**Keywords:** inflammatory bowel disease, ferroptosis, ACSL4, intestinal barrier disruption, antioxidant

## Abstract

**Background:**

The pathogenesis of inflammatory bowel disease (IBD) is closely associated with the dysfunction of the intestinal epithelial barrier, leading to increased bacterial translocation, leukocyte infiltration, and mucosal injury, which may act as a pivotal or incipient event in the pathophysiology of the disorder. The primary objective of this study is to examine the key genes implicated in IBD and the perturbation of intestinal epithelial cell function.

**Methods:**

The genes associated with ferroptosis were identified through the utilization of the Gene Expression Omnibus (GEO) database and the GeneCard database. Additionally, an *in vitro* model of IBD was established by stimulating Caco-2 cells with lipopolysaccharides (LPSs) to investigate the molecular mechanisms underlying intestinal epithelial cell dysfunction.

**Results:**

We discovered evidence that establishes a connection between ferroptosis and the inflammatory responses associated with the development of IBD. This evidence suggests that IBD patients who exhibit an inflammatory response have higher expression of the acyl-CoA synthetase long-chain family member 4 (ACSL4) gene compared to IBD patients without an inflammatory response or healthy individuals. Exposure to LPS at concentrations of 1 or 10 μg/mL resulted in a significant upregulation of ferroptosis-related genes ACSL4, GPX4, and SLC7A11, as well as an increase in ferroptosis biomarkers MDA and a decrease in CAT and GSH-Px levels compared to the control group. Inhibition of ACSL4 using si-ACSL4 or rosiglitazone demonstrated protective effects against LPS-induced ferroptosis and NF-κB-mediated inflammatory response.

**Conclusion:**

ACSL4 shows potential as a promising target for ferroptosis in the prevention and treatment of IBD and dysfunction of intestinal epithelial cells.

## 1 Introduction

Inflammatory bowel disease (IBD), encompassing Crohn’s disease (CD) and ulcerative colitis (UC), manifests as fluctuating episodes of chronic inflammation in distinct regions of the gastrointestinal tract, resulting in symptoms such as diarrhea and abdominal pain [1]. The inflammatory process is initiated by a cell-mediated immune reaction within the gastrointestinal mucosa, while the precise etiology of IBD remains elusive. There is an indication that dysregulated immune responses triggered by commensal gut microbiota in individuals with a complex genetic susceptibility may contribute to the pathogenesis of IBD [2,3,4]. Based on projections from the 2020 United States Census, it is estimated that approximately 2.39 million individuals in the United States have been diagnosed with IBD, and the prevalence of IBD per 100,000 population was calculated to be 10.9 (95% CI, 10.6–11.2) [5]. In China, the incidence of IBD in 2016 was determined to be 10.04 (95% confidence interval, 6.95–13.71) per 100,000 person-years [6]. The development of IBD is intricately linked to the impairment of intestinal epithelial barrier functions, resulting in heightened bacterial translocation, infiltration of leukocytes, and mucosal damage, potentially serving as an initiating or early event in the progression of the disease [2,7]. Lipopolysaccharide (LPS), also referred to as endotoxin, is a significant factor in the development of infectious diseases and is primarily responsible for the associated morbidity and mortality [8]. Typically, the increase in LPS levels resulting from the proliferation of Gram-negative bacteria, combined with the accumulation of inflammatory or immune cytokines, contributes to the initiation and progression of IBD by promoting intestinal epithelial cell death, translocation of intestinal luminal contents, commensal microbiota, and pathogenic microbes into the gut lamina propria [9,10,11]. Hence, the preservation of the integrity of the intestinal epithelium plays a crucial role in upholding intestinal homeostasis through the prevention of translocation of gut microbiota or detrimental exogenous factors. Consequently, the impairment of this protective barrier leads to the initiation and advancement of IBD. Nevertheless, the identification of the principal genes responsible for IBD and the disruption of the intestinal barrier induced by LPS remains elusive.

Research has demonstrated that individuals with IBD may exhibit key characteristics of ferroptosis in their damaged gut, including iron accumulation, glutathione (GSH) depletion, glutathione peroxidase (GSH-Px) inactivation, and lipid peroxidation [12,13]. Furthermore, studies have indicated that high dietary iron intake is associated with an increased risk of developing UC and exacerbating clinical symptoms in UC patients, with reactive oxygen species (ROS) production in UC mucosa correlating with disease severity [14]. Treatment with iron-chelating agents, which inhibit ferroptosis, has been shown to reduce ROS production and ameliorate intestinal inflammation [12,13,15], suggesting a potential connection between IBD and ferroptosis. Acyl-CoA synthetase long-chain family member 4 (ACSL4) is an enzymatic catalyst responsible for the conversion of fatty acids into fatty acyl-CoA esters, thereby regulating the biosynthesis of lipids [16]. ACSL4 has been identified as a crucial factor in the occurrence of ferroptosis, and inhibiting ACSL4 through genetic or pharmacological means has been shown to offer cellular protection against lipid peroxidation and ferroptosis [16,17,18]. Previous research has indicated that ACSL4-mediated ferroptosis is implicated in various pathological conditions, including acute kidney injury [17,19], cerebral ischemia/reperfusion injury [20,21], brain injury, and neuroinflammation [3,22,23,24]. ACSL4 has been observed to be upregulated in the ileum and colon of patients diagnosed with CD and UC, as well as in mice with experimentally induced colitis using dextran sulfate sodium [25,26]. Additionally, ACSL4 activation has been implicated in contributing to tissue injury caused by ferroptosis in intestinal ischemia/reperfusion (I/R), as reported by Li et al. [27]. This activation is believed to be mediated, at least partially, by the transcription factor special protein 1, which enhances ACSL4 transcription by binding to the ACSL4 promoter region [27]. Additionally, it has been observed that the silencing of ACSL4 through siRNA also provides protection to Caco-2 cells against hypoxia/reoxygenation-induced lipid peroxidation and cell death [27]. However, the impact of ACSL4 in LPS-induced intestinal barrier disruption remains uncertain.

In our research, we noted a marked increase in ACSL4 gene expression in the inflamed intestinal tissues of patients diagnosed with CD and UC in comparison to the non-inflamed intestinal tissues of individuals with CD and UC. This observation suggests a potential involvement of the inflammatory response in the activation of ACSL4 expression during the advancement of IBD. Furthermore, we developed an *in vitro* model of IBD utilizing LPS-stimulated Caco-2 cells to evaluate the possible therapeutic effects of genetic or pharmacological inhibition of ACSL4 on intestinal epithelial cell dysfunction.

## 2 Materials and methods

### 2.1 Differentially expressed gene (DEG) screening

The Gene Expression Omnibus (GEO) database with GSE95095 and GSE179285 datasets were utilized to screen DEGs based on |Log2(fold change)| > 2 and p.adj < 0.05. R software (version 4.2.1) with GEOquery package (version 2.64.2), limma package (version 3.52.2), ggplot2 package (version 3.3.6), and ComplexHeatmap package (version 2.13.1) were used to analyze the GEO dataset, as described previously [28].

### 2.2 Ferroptosis-related genes in IBD

The GeneCards database (https://www.genecards.org/) provides comprehensive information about human genes. Ferroptosis-associated genes were downloaded from the GeneCards database. We obtained the ferroptosis-related genes in IBD by intersecting the genes that were DEGs in the GSE95095 dataset and GeneCards database. Venn diagram was visualized using R software (version 4.2.1) with ggplot2 package (version 3.3.6) and VennDiagram package (version 1.7.3).

### 2.3 Cell culture and transfection

Caco-2 was purchased from Wuhan Pricella Biotechnology Co., Ltd (Wuhan, China), and the cells were cultured in Caco-2-complete medium (DMEM supplemented with 10% FBS, 1% NEAA, and 1% penicillin–streptomycin mixed solution incubated at 37°C in a humidified incubator with 5% CO_2_. Caco-2 cells were stimulated with LPS at concentrations of 1 or 10 μg/mL or rosiglitazone (RSG; 100 μM; Cat. no: HY-17386, MedChenExpress), which is considered a potential ferroptosis inhibitor. The small interfering RNAs (si-RNAs), including si-Con and si-ACSL4, were synthesized by Gene-Pharma (Shanghai, China). Lipofectamine 3000 (Invitrogen) was used for cell transfection according to the manufacturer’s instructions.

### 2.4 Cell viability

CCK-8 kit (Beyotime Institute of Biotechnology, Haimen, China) was used to measure cell proliferation *in vitro*. In brief, Caco-2 cells were transferred to 96-well plates and co-culture with CCK-8 solution at 37°C for 2 h, and the absorbance was tested with a microplate reader at 450 nm.

### 2.5 Measurements of biochemical parameters and cell apoptosis

Malondialdehyde (MDA; Cat. no: A003-1-2), catalase (CAT; Cat. no: A007-1-1), GSH-Px (Cat. no: A005-1-2), tumor necrosis factor-α (TNF-α; Cat. no: H052-1-2), and interleukin-1β (IL-1β; Cat. no: H002-1-2) in the supernatant liquid and cells were detected using the commercial kit from Nanjing Jiancheng Bioengineering Institute (Nanjing, China). IL-6 ELISA kit (Cat. no: E-EL-H6156) was obtained from Elabscience (Wuhan, China). Cell apoptosis was analyzed by flow cytometry with Annexin V-FITC/PI (Beyotime Institute of Biotechnology, Haimen, China), as described previously [29].

### 2.6 RT-qPCR

Standard RT-qPCR procedures were performed, as described previously [30]. Briefly, total RNA was extracted using TRIzol^®^ (Invitrogen; Thermo Fisher Scientific, Inc., Waltham, MA, USA). Moloney murine leukemia virus reverse transcriptase (Invitrogen; Thermo Fisher Scientific, Inc.) and TaqMan^®^ Universal PCR Master Mix (Thermo Fisher Scientific, Inc.) were utilized for RT-qPCR. PCR primers were synthesized by Sangon Biotech (Shanghai, China).

### 2.7 Western blot

Total protein preparation and the procedures of western blot were performed as described previously [31]. The primary antibody for NF-κB/p65 (cat. no: #8242; dilution ratio 1:1,000) and horseradish peroxidase-conjugated secondary antibody (anti-rabbit IgG-HRP; cat. no: #7040) were obtained from Cell Signaling Technology. Protein bands were visualized using an enhanced chemiluminescence kit (Thermo Fisher Scientific, Inc.). Signals were analyzed with Quantity One^®^ software version 4.5 (Bio Rad Laboratories, Inc., Hercules, CA, USA). Anti-histone (Cat. no: 7631; Cell Signaling Technology, MA, USA, 1:1,000) was used as the control antibody.

### 2.8 Statistical analysis

Data are presented as mean ± standard deviation. Statistical analysis was performed using IBM SPSS Statistics Version 23.0 (SPSS Inc., Chicago, IL, USA). Inter-group differences were analyzed using one-way ANOVA. A *P*-value < 0.05 indicates a statistically significant difference.

## 3 Results

### 3.1 DEGs in patients with CD

**Figure 1:**
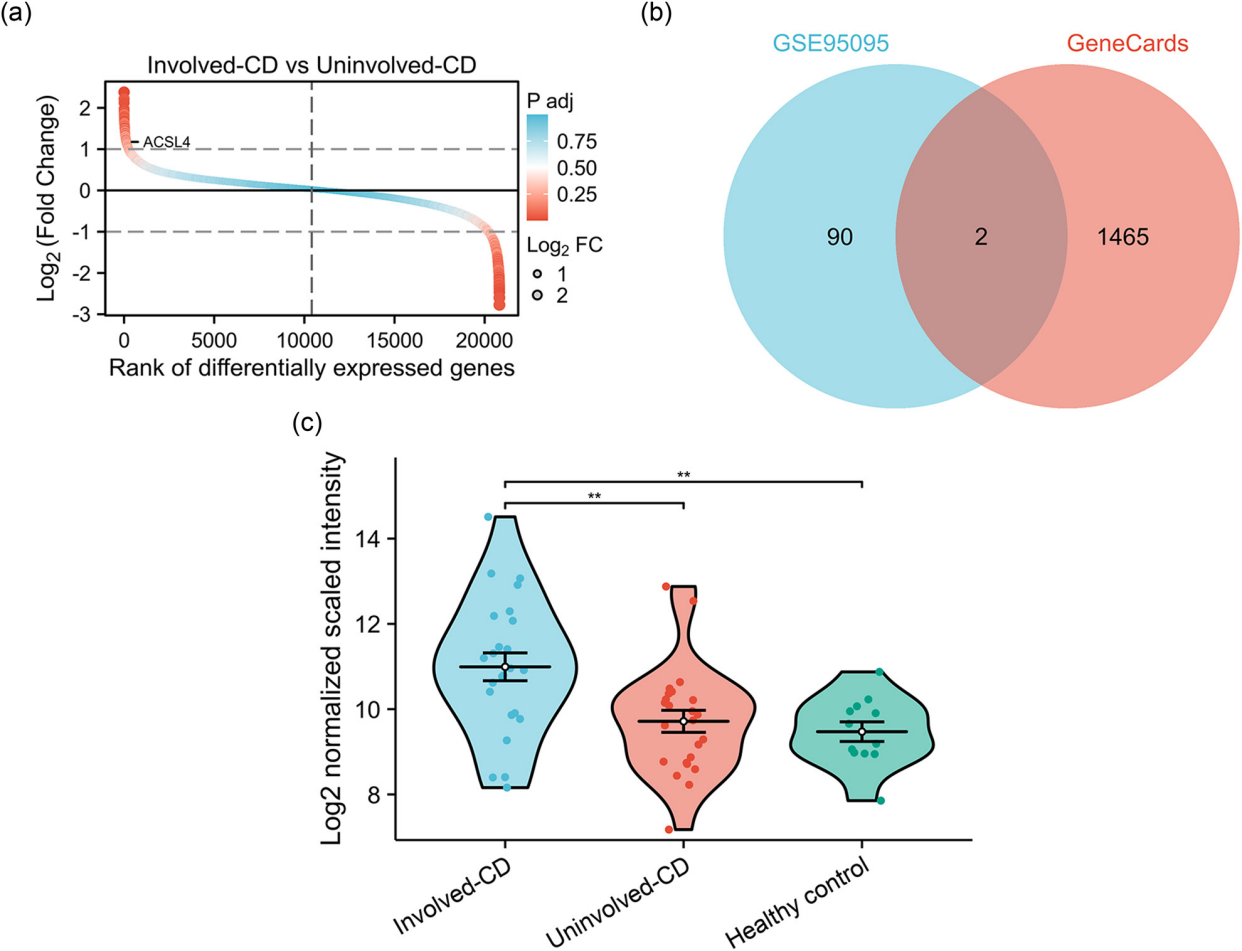
DEGs in patients with CD. In the dataset GSE95095, CD tissues (involved-CD; *n* = 24) and adjacent non-CD tissues (uninvolved-CD; *n* = 24) were used to identify DEGs with |LogFC| > 1 and p. adj < 0.05 (a). To identify genes related to ferroptosis, we utilized the GeneCard databases (https://www.genecards.org/) to search “ferroptosis” as the keyword and downloaded the list of the FAGs, which combined with DEGs in CD patients to obtain FAGs in the progression of CD (b). ACSL4 gene expression in intestinal tissues of CD tissues (involved-CD; *n* = 24), adjacent non-CD tissues (uninvolved-CD; *n* = 24) and healthy controls (*n* = 12) was analyzed using GEO DataSet of GSE95095 (c); ^**^ *P* < 0.01. DEGs, differentially expressed genes; FAGs, ferroptosis-associated genes.

To analyze DEGs in the progression of CD, we performed differential expression analysis between CD tissues (involved-CD; *n* = 24) and adjacent non-CD tissues (uninvolved-CD; *n* = 24) with |LogFC| > 1 and p. adj < 0.05. In the dataset GSE95095, a total of 92 DEGs, comprising 36 upregulated genes and 56 downregulated genes, were identified in CD tissues compared to non-CD tissues (Figure 1a). To identify genes related to ferroptosis, we utilized the GeneCard databases (https://www.genecards.org/) to search “ferroptosis” as the keyword and downloaded the list of the ferroptosis-associated genes (FAGs), and ACSL4 (Log2FC = 1.178; p.adj = 0.034) and MIR626 (Log2FC = −1.184; p.adj = 0.034) might be FAGs in the progression of CD (Figure 1b). Compared with both healthy controls and uninvolved-CD tissues, ACSL4 gene expression was significantly increased in involved-CD tissues (Figure 1c).

### 3.2 ACSL4 gene expression was significantly upregulated in CD-inflamed and UC-inflamed tissues of patients with IBD

**Figure 2:**
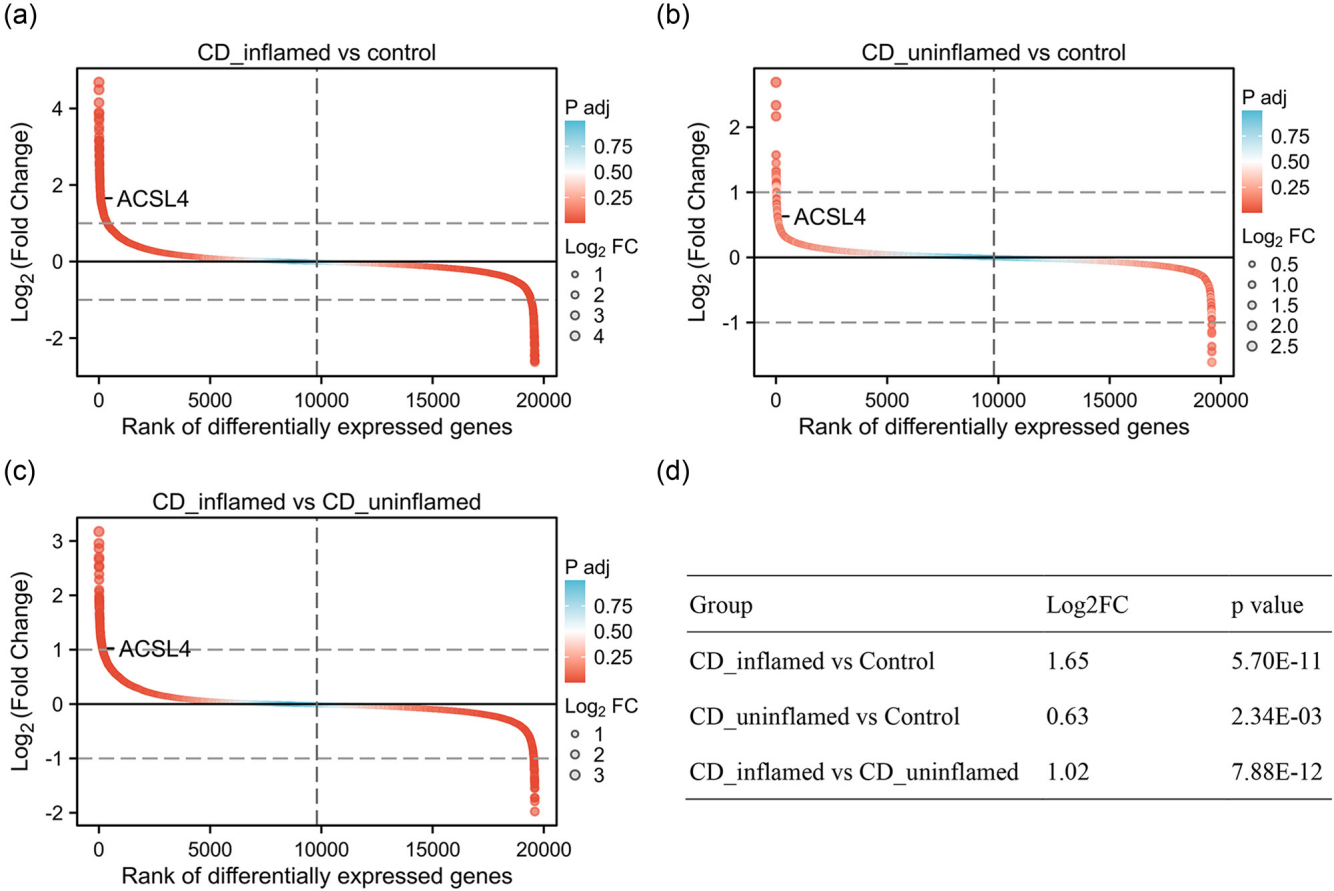
ACSL4 gene expression in CD-inflamed patients. ACSL4 gene expression was analyzed by comparison between any two means using GEO DataSet (GSE179285) as follows: CD-inflamed group vs control group (a) and (d), CD-uninflamed group vs control group (b) and (d), and CD-inflamed group vs CD-uninflamed group (c) and (d).

**Figure 3:**
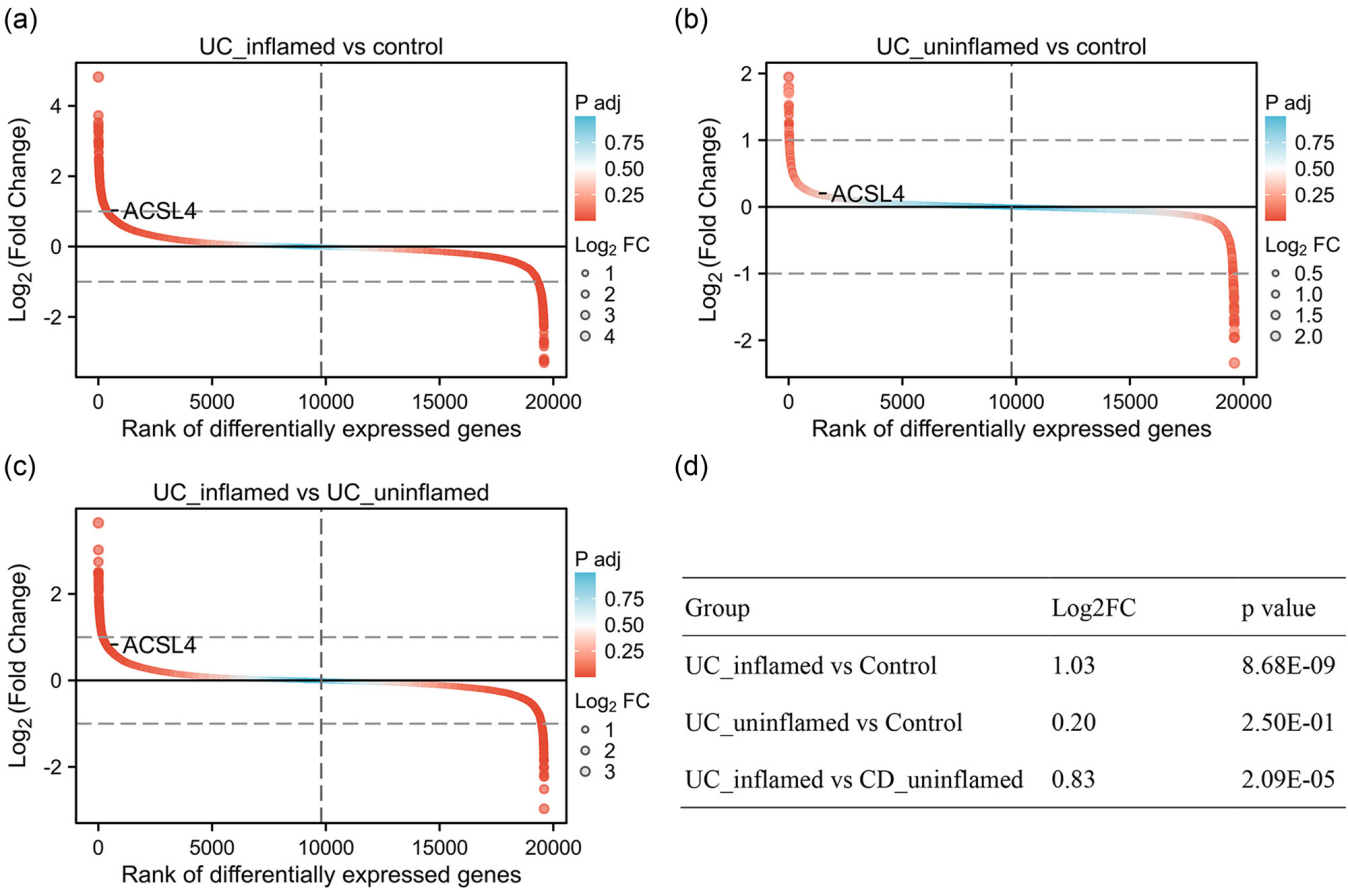
ACSL4 gene expression in UC-inflamed patients. ACSL4 gene expression was analyzed by comparison between any two means using GEO DataSet (GSE179285) as follows: UC-inflamed group vs control group (a) and (d), UC-uninflamed group vs control group (b) and (d), and UC-inflamed group vs UC-uninflamed group (c) and (d).

To confirm the expression of ACSL4 in IBD patients, the dataset GSE179285, including 31 healthy controls, 47 CD-inflamed, 121 CD-uninflamed, 23 UC-inflamed, and 32 UC-uninflamed patients, was used to evaluate ACSL4 gene expression in the intestinal tissue. As shown in Figure 2, ACSL4 was significantly elevated in CD-inflamed group vs control group (Log2FC = 1.65; p.adj = 5.70 × 10^−11^; Figure 2a and d), CD-uninflamed group vs control group (Log2FC = 0.63; p.adj = 2.34 × 10^−3^; Figure 2b and d), and CD-inflamed group vs CD-uninflamed group (Log2FC = 1.03; p.adj = 7.88 × 10^−12^; Figure 2c and d). As shown in Figure 3, ACSL4 was significantly elevated in UC-inflamed group vs control group (Log2FC = 1.03; p.adj = 8.68 × 10^−9^; Figure 3a and d), UC-uninflamed group vs control group (Log2FC = 0.20; p.adj = 2.50 × 10^−1^; Figure 3b and d), and UC-inflamed group vs UC-uninflamed group (Log2FC = 0.83; p.adj = 2.09 × 10^−5^; Figure 3c and d). These findings suggest that IBD patients with inflammatory response might be associated with higher ACSL4 gene expression than IBD patients without inflammatory response or healthy controls.

### 3.3 Si-ACSL4 or RSG treatment reverses LPS-induced ferroptosis in intestinal epithelial cells

**Figure 4:**
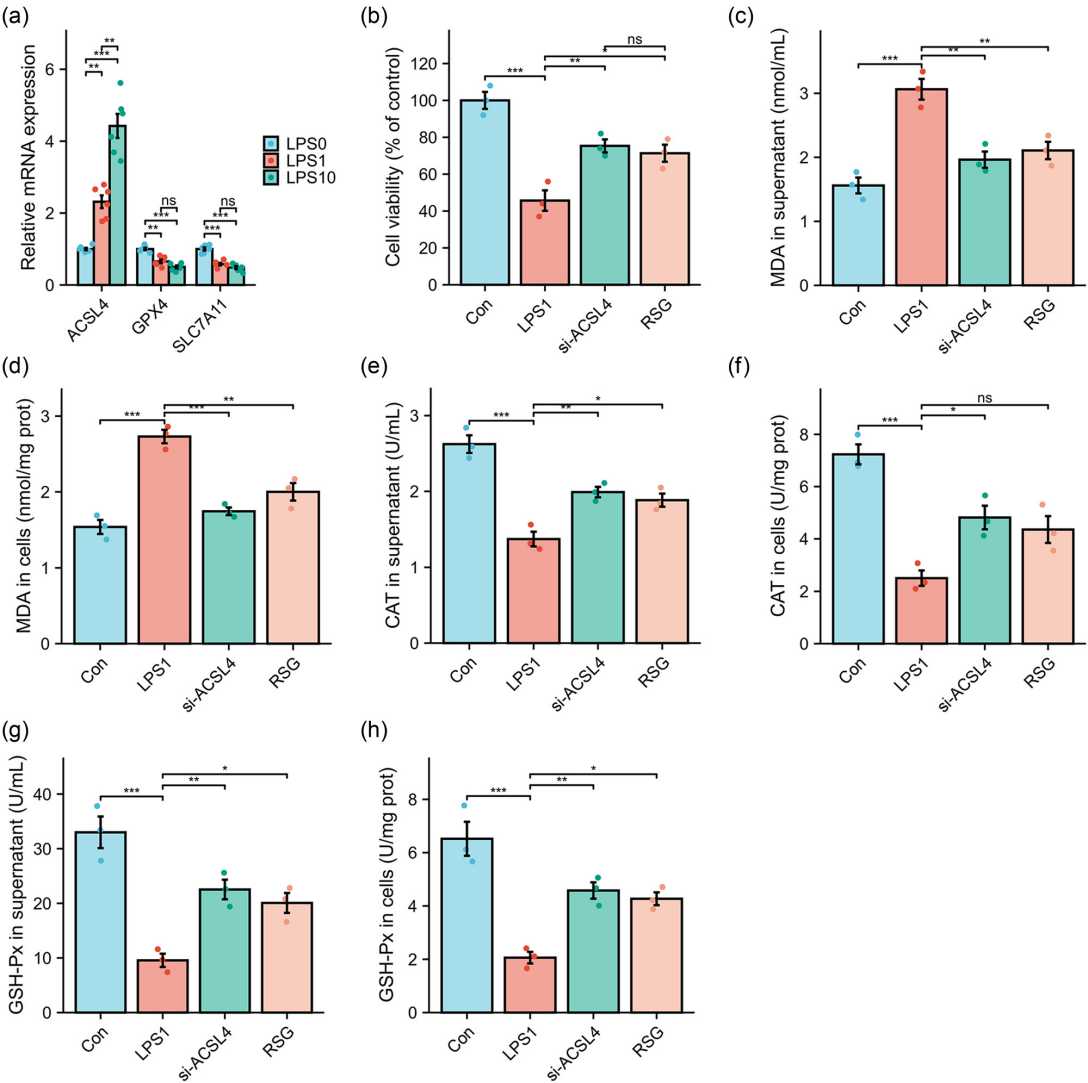
Si-ACSL4 or RSG treatment reverses LPS-induced ferroptosis in intestinal epithelial cells. LPS (1 or 10 μg/mL) exposure to Caco-2 cells for 24 h, the gene expression of ACSL4, GPX4, and SLC7A11 was measured using RT-qPCR (a). LPS (1 μg/mL) exposure to Caco-2 cells for 24 h with or without si-ACSL4 or RSG treatment, cell viability was evaluated using CCK-8 assays (b); the production of MDA in the supernatant (c) and cells (d) was measured using thiobarbituric acid method; CAT (e) and (f) and GSH-Px (g) and (h) in the supernatant and cells were measured using ELISA kits. ^*^ *P* < 0.05; ^**^ *P* < 0.01; ^***^ *P* < 0.001; ns, no significant.

In order to examine the potential role of LPS in inducing intestinal barrier disruption through the activation of ferroptosis, the expression levels of key regulators of ferroptosis were assessed in Caco-2 cells treated with LPS. Caco-2 cells, a well-established human intestinal epithelial cell line, are commonly employed as a cellular model to investigate the mechanisms underlying intestinal barrier disruption when stimulated with LPS [32]. As shown in Figure 4a, the exposure to LPS at concentrations of 1 or 10 μg/mL resulted in a significant upregulation of the gene expression of ACSL4, GPX4, and SLC7A11 compared to the control group. Furthermore, treatment with LPS at a concentration of 1 μg/mL led to a substantial decrease in cell viability, which was reversed by si-ACSL4 or RSG treatment, effectively counteracting the inhibitory effects of LPS on cell growth (Figure 4b). The levels of MDA, a marker of membrane damage, were significantly increased by LPS treatment in both the supernatant (Figure 4c) and cells (Figure 4d) compared to the control group. However, the inhibition of ACSL4 with Si-ACSL4 or RSG resulted in a reduction in the production of MDA, indicating a decrease in membrane damage. Additionally, the expression of CAT (Figure 4e and f) and GSH-Px (Figure 4g and h) in both the supernatant and cells of LPS-treated Caco-2 cells was significantly decreased. However, this down-regulation was reversed when si-ACSL4 or RSG was administered.

### 3.4 Effect of si-ACSL4 or RSG on ferroptosis-related inhibiting factors

**Figure 5:**
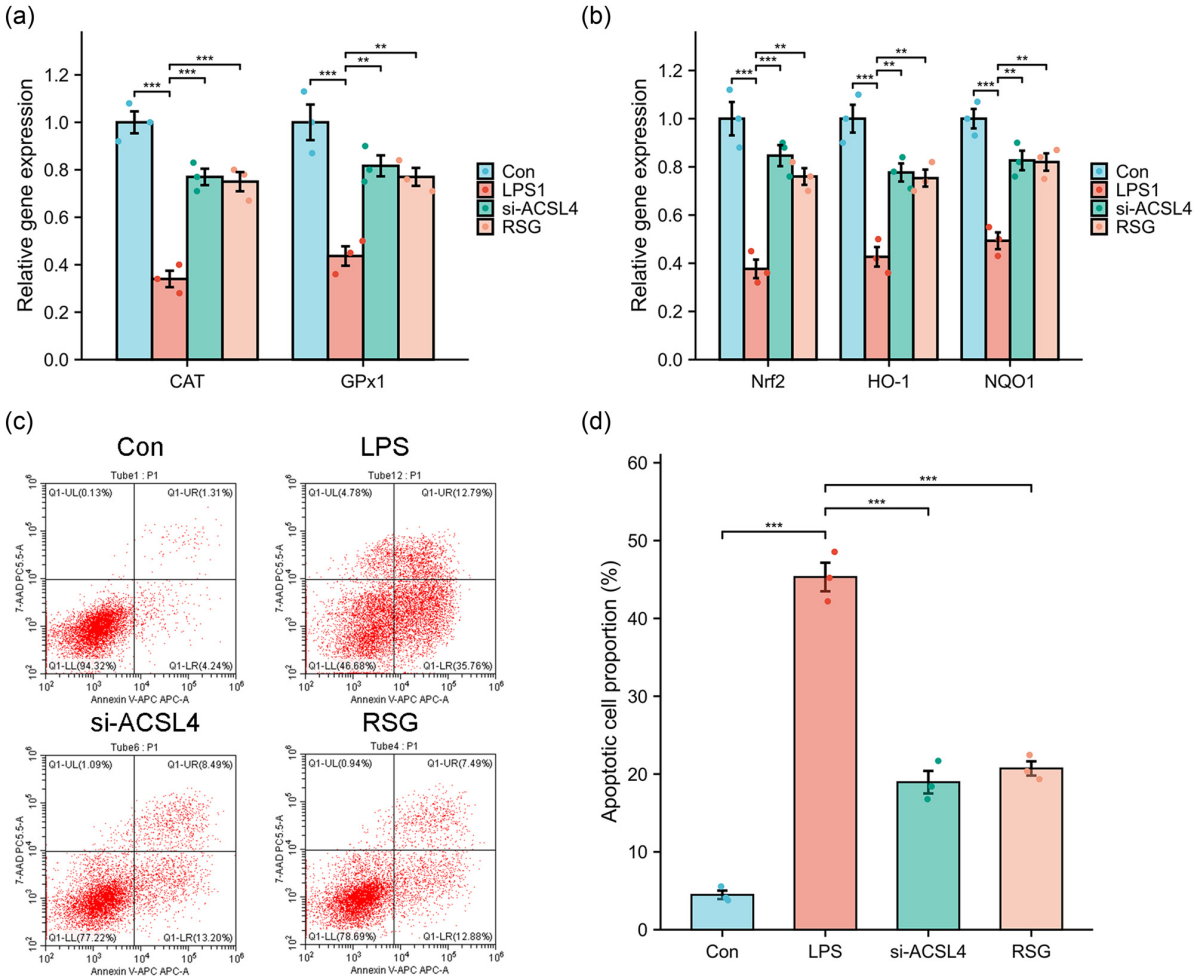
The effect of si-ACSL4 or RSG on ferroptosis-related inhibiting factors. The gene expression of antioxidases (CAT and GPx1; (a) and Nrf2 signaling components (Nrf2, HO-1, and NQO1; (b) was measured using RT-qPCR in LPS-treated Caco-2 cells with or without si-ACSL4 or RSG treatment; the percentage of apoptotic cells was evaluated using Annexin V-FITC/PI kit (c) and (d). ^**^ *P* < 0.01; ^***^ *P* < 0.001.

The gene expression levels of antioxidases and components of the Nrf2 signaling pathway were assessed in Caco-2 cells treated with LPS. The transcription factor known as nuclear factor erythroid 2-related factor 2 (NRF2) serves as a crucial regulator of the cellular antioxidant response, governing the expression of genes that counteract oxidative and electrophilic stresses. These genes play a vital role in mitigating lipid peroxidation and ferroptosis [33]. Figure 5a and b demonstrates that the downregulation of CAT, GPx1, Nrf2, HO-1, and NQO1 mRNA levels induced by LPS was reversed upon administration of si-ACSL4 or RSG. Moreover, the percentage of apoptotic cells in the LPS group (45.32 ± 2.60%) was significantly higher compared to the control group (4.48 ± 0.77%). Conversely, treatment of Caco-2 cells with si-ACSL4 (18.95 ± 2.05%) or RSG (20.72 ± 1.29%) significantly reduced the proportion of LPS-induced apoptotic cells (Figure 5c and d).

### 3.5 Effect of si-ACSL4 or RSG on LPS-induced inflammation

**Figure 6:**
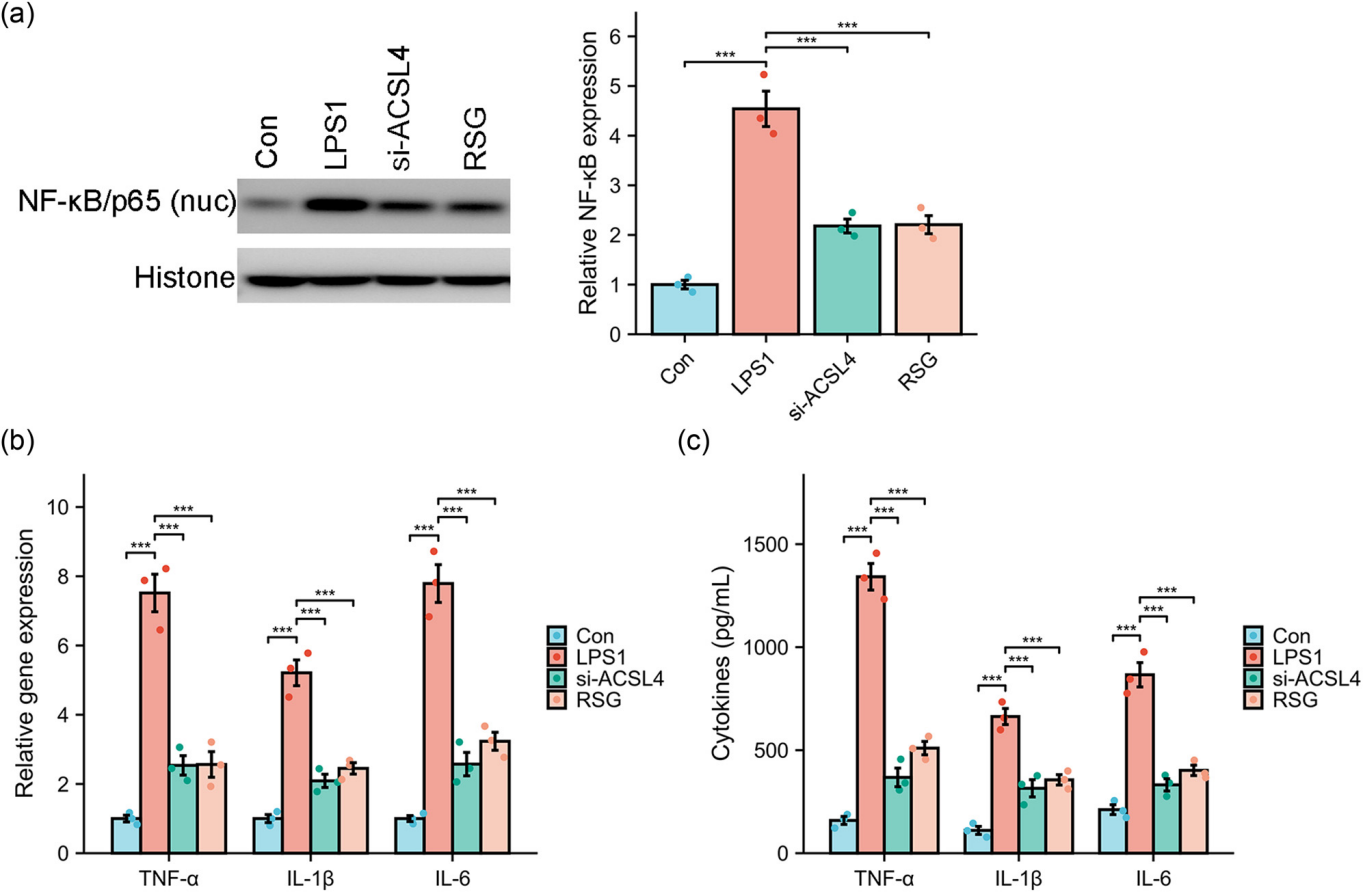
The effect of si-ACSL4 or RSG on LPS-induced inflammation. LPS (1 μg/mL) exposure to Caco-2 cells for 24 h with or without si-ACSL4 or RSG treatment, the protein expression of NF-κB/p65 was measured using western blot (a); the gene expression (b) and production in the supernatant (c) of TNF-α, IL-1β and IL-6 were analyzed using RT-qPCR and ELISA kit, respectively. ^***^ *P* < 0.001.

**Figure 7:**
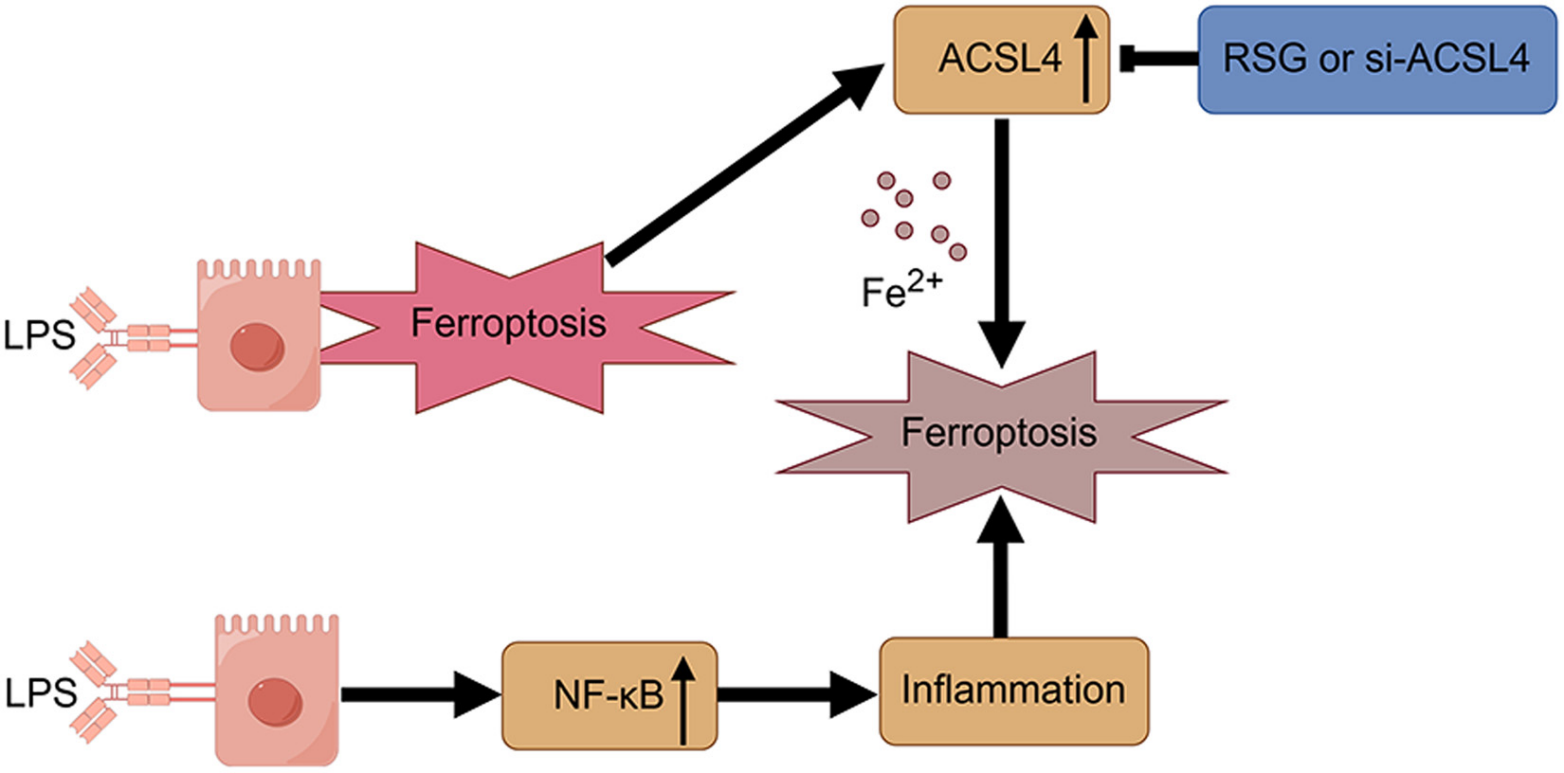
Schematic diagram of proposed effects of ferroptosis and inflammation in LPS-induced intestinal barrier disruption. The induction of ACSL4 expression and inhibition of antioxidant reaction and Nrf2 signaling pathway by LPS contribute to the development of ferroptosis and inflammation in the context of intestinal barrier disruption. LPS, lipopolysaccharide; NF-κB, nuclear factor kappa-B; GPX4, glutathione peroxidase 4; ACSL4, acyl-CoA synthetase long-chain family member 4; RSG, rosiglitazone.

The TLR4/NF-κB signaling pathway is recognized as the primary mechanism responsible for the production of inflammatory cytokines induced by LPS [34]. In this study, we observed a significant increase in the protein expression of NF-κB/p65 in the cell nucleus (Figure 6a), as well as the gene expression (Figure 6b) and production in the supernatant (Figure 6c) of TNF-α, IL-1β, and IL-6 in LPS-stimulated Caco-2 cells. However, the overactivation of the inflammatory response in Caco-2 cells induced by LPS was effectively hindered by the treatment of si-ACSL4 or RSG. According to the findings above, it can be concluded that LPS triggers a significant upregulation of ACSL4 while simultaneously inhibiting antioxidant reactions and the Nrf2 signaling pathway. Consequently, targeting ACSL4 may hold promise as a potential therapeutic approach for preventing and treating LPS-induced IBD, as depicted in Figure 7.

## 4 Discussion

In our study, we found evidence linking ferroptosis to the inflammatory responses associated with the development of IBD. Specifically, we observed a positive correlation between the upregulation of ACSL4 expression and the presence of inflamed CD and UC in human subjects. *In vitro* experiments revealed ACSL4 as a notable factor in the impairment of the intestinal epithelial cell resulting from LPS stimulation. Additionally, our study has shown that the inhibition of ACSL4 through si-ACSL4 or RSG can offer effective protection against LPS-induced intestinal epithelial injury. Our results suggest that targeting ACSL4 may hold promise for the management and mitigation of LPS-induced IBD.

IBD is frequently linked to chronic inflammation of the intestines, which is accompanied by dysbiosis of the intestinal microenvironment and epithelial cell death [35]. Recent studies have elucidated the presence of dysregulated cell death in inflamed regions, which further compromises the integrity of the intestinal barrier and intensifies the inflammatory response [36,37]. Ferroptosis, a recently identified form of regulated cell death, is initiated by the buildup of lipid peroxides catalyzed by intracellular free iron. The essential features of ferroptosis, including iron accumulation, depletion of GSH, inactivation of GPX4, and lipid peroxidation, have been extensively documented in the damaged intestinal tissue of patients diagnosed with IBD [37,38]. In the DSS-induced experimental model of UC, ferroptosis was facilitated through the activation of endoplasmic reticulum stress signaling, ultimately resulting in the death of intestinal epithelial cells [39]. In our experimental model of LPS-induced IBD, we observed a reduction in GPX4 expression and antioxidant products within Caco-2 cells. Additionally, an augmentation in the ratio of apoptotic cells and the synthesis of inflammatory cytokines was observed in Caco-2 cells stimulated with LPS. Our discoveries suggest that ACSL4, serving as an indicator of ferroptosis, experienced upregulation and played a role in the LPS-induced death of epithelial cells and the ensuing inflammatory reaction.

ACSL4 was found to be upregulated in ischemic intestinal tissues of both humans and mice compared to normal tissues. However, treatment with the ACSL4 inhibitor, RSG or si-ACSL4, showed a protective effect against intestinal I/R injury by restoring GPX4 expression, reducing COX2 expression, and decreasing lipid peroxidation, as evidenced by decreased levels of 12-HETE, 15-HETE, 5-HETE, and lactate dehydrogenase [27]. However, the precise relationship between ACSL4 and inflammation-related intestinal epithelial cell damage has yet to be determined. A previous study demonstrated that ACSL4 expression was upregulated in microglia during LPS-induced inflammation, and knockdown of ACSL4 was found to mitigate neuroinflammation by inhibiting NF-κB signal transduction [3]. Additionally, LPS-stimulated hippocampus cells were found to induce ferroptosis in the hippocampus, as evidenced by increased levels of ROS, iron content, and MDA, as well as decreased levels of GSH. Furthermore, the expression of ferroptosis-related proteins (GPX4, ACSL4, and SLC7A11) was found to be altered [40]. The study demonstrated an increase in ACSL4 expression in an *in vitro* colitis model using LPS-stimulated Caco-2 cells and immune cells from individuals with UC [25,41]. Additionally, studies have indicated that ACSL4 may be involved in immune infiltration, triggering inflammatory reactions and causing oxidative stress during the progression of IBD [41,42,43]. Tan et al. [41] found that ACSL4 was significantly upregulated in immune cells in UC. The ERK-cPLA2-ACSL4 axis was identified as mediating M2 macrophage ferroptosis, which hinders mucosal healing in UC. Treatment with the ferroptosis inhibitor Fer-1 resulted in a notable decrease in ferroptosis in macrophages within the colon tissue, accompanied by an increase in the proportion of M2 macrophages. This suggests that targeting ferroptosis in M2 macrophages may represent a promising therapeutic approach for managing UC [42]. ACSL4 was shown to mediate ferroptosis in both *in vivo* and *in vitro* classic UC models by activating inflammation and oxidative stress [43]. Our findings indicate that treatment with RSG or si-ACSL4 effectively mitigated LPS-induced ferroptosis and inflammation in Caco-2 cells, suggesting that ACSL4 inhibition holds promise as a potential therapeutic approach for ameliorating LPS-induced disruption of the intestinal epithelial barrier.

Although we have identified ACSL4 as a potential target for preventing and treating IBD, our research has some limitations. First, we did not collect enough clinical samples to demonstrate the expression and molecular mechanisms of genes related to iron-induced cell death. However, we can guarantee that future studies will explore the expression and molecular mechanisms of genes associated with iron-induced cell death in clinical samples. Second, we have not examined the upstream and downstream signaling pathways linked to ACSL4 or validated them in relevant animal models.

In summary, our study demonstrates that the inhibition of ACSL4 effectively mitigates the disruption of the intestinal barrier induced by LPS through the suppression of both ferroptosis and inflammation. Moreover, our findings suggest that the inflammatory response could potentially serve as a triggering factor for the upregulation of ACSL4 expression in patients with IBD, encompassing both CD and UC. These results offer valuable insights for the advancement of innovative therapeutic strategies aimed at alleviating inflamed IBD and epithelial injury associated with ferroptosis, by suppressing ACSL4 expression.
